# Evaluation and improvement of two European soybean VCU networks

**DOI:** 10.1007/s00122-026-05282-x

**Published:** 2026-05-25

**Authors:** Jip J. C. Ramakers, Waqas A. Malik, Kleens Mechtler, Christine Fintz, Cécile Collonnier, François Laurens, Hans-Peter Piepho, Fred A. van Eeuwijk

**Affiliations:** 1 Mathematical and Statistical Methods group–Biometris, Wageningen University and Research, Wageningen, the Netherlands; 2 Biostatistics Unit, Institute of Crop Science, University of Hohenheim, Stuttgart, Germany; 3 Austrian Agency for Health and Food Safety (AGES), Vienna, Austria; 4 Variety Study Department, French Variety and Seed Study and Control Group (GEVES), Beaucouzé, France; 5 Community Plant Varieties Office (CPVO), Angers, France; 6 University of Angers, INRAE, Institut Agro, IRHS, SFR QuaSaV, Angers, France

## Abstract

Soybean (*Glycine max*, (L.) Merr.) productivity gains depend on accurate estimates of genetic trends, genotypic effects (*G*), and genotype-by-environment interactions (GEI). Here, we compiled a Value for Cultivation and Use (VCU) database of soybean varieties, evaluated from 2003 to 2018 in Austria (AT) and France (FR), to estimate genetic trends, *G* and GEI magnitudes, and variety-testing precision in grain yield, protein yield and protein content (%), using mixed-model methodology. We found that *G* and GEI variances were comparable in both countries, with a variety-testing precision, expressed as a relative detection threshold (*LSD* for elite standards), of about 7.5% for grain yield and protein yield, and about 2% for protein content. Grain yield correlated moderately negatively with protein content at the genotypic level. Despite this, although genetic trends in protein content were non-significant and near-zero (slightly negative in FR), trends in grain yield and protein yield were significant and strongly positive (1.97–2.05% yr^−1^, AT; 0.75–0.86% yr^−1^, FR). Given the high heritability of protein content (> 0.9), there is scope for selection, which may come at the expense of grain yield. Both networks appeared well optimized in terms of the number of test locations, but gains in precision could be obtained from adding an extra test year. Combining data from both VCU networks enabled effective comparison between varieties not jointly tested. Such cross-country comparison could be further improved by strategic use of check varieties, genetic markers and environmental covariates to enhance genotypic and environmental connectivity between networks.

**Supplementary Information:**

The online version contains supplementary material available at 10.1007/s00122-026-05282-x.

## Introduction

Soybean (*Glycine max*, (L.) Merr.) is one of the most important crops for oil and protein, with worldwide seed production almost tripling in the past three decades up to 371 Mt in 2023 (FAO 2025). Although the European Union (EU) largely relies on import—mainly for animal feed purposes (Tillie & Rodríguez-Cerezo 2015)—soybean is one of the three major oil/protein crops grown within the EU, producing 2–3 Mt annually over the past decade (Eurostat 2025). To tackle rising protein demands while at the same time combat concerns about land-use conversion (Morton et al. 2006) and the environmental impact of soy production (Sun et al. 2018), it is imperative to increase productivity per unit area. In this context, a European research initiative was launched in 2019 to increase efficiency in European variety testing across numerous crops (H2020 INVITE; https://www.h2020-invite.eu/). Among participating countries, Austria and France play leading roles in EU soybean production, making robust variety evaluation particularly critical in these countries.

Progress in crop production relies on the release of improved commercial varieties. Under European regulations (Council Directives 2002/53/EC and 2003/90/EC), candidate varieties must meet Distinctness, Uniformity, and Stability (DUS) requirements to be listed at the national or EU level (CPVO 2019). Simultaneously, they are assessed for their Value for Cultivation and Use (VCU) by national Examination Offices (EOs) through multi-environment trials (METs) conducted over two to three years. VCU evaluations consider traits such as grain yield by maturity or quality class, disease resistance, end-use value, and tolerance to abiotic stress (Bundessortenamt 2003; Raad voor Plantenrassen 2022). VCU-MET data exhibit a ‘diagonal’ occurrence structure over time: as new varieties enter and others leave the system every year, there is a high overlap in varieties between consecutive years and lower overlap between distant years, with only ‘check’ or ‘standard’ varieties providing continuity across longer time spans.

Since VCU evaluation typically spans two to three years, EOs capture only a snapshot of environmental conditions to assess variety performances. However, historical VCU data provide an opportunity to evaluate network efficiency and quantify genetic improvement in key agronomic traits over time (Ramakers et al. 2026). Trialling precision enables genetic gain by allowing selection to distinguish superior varieties from the background noise of genotype-by-environment interaction (GEI) variance, although actual gain is ultimately bounded by available genetic variation (Piepho et al. 2022). Evaluation of current trialling networks for genetic gain and precision therefore begins with quantifying GEI variation in key traits from historical data. Key traits for soybean include seed or grain yield (e.g. in t ha^−1^) and protein yield/content (e.g. in absolute terms in t ha^−1^ or in relative terms in g kg^−1^ yield). Protein content is particularly interesting as it shows a negative genetic correlation with yield (de Felipe et al. 2016; Milioli et al. 2022; Morrison et al. 2000). Genetic trends in these key traits have been widely estimated for soybean, mainly in the USA and South-America (see Table 3 for a summary), but are lacking for EU systems. Moreover, a systematic evaluation of VCU-testing systems, as well as an investigation into alternative configurations, is completely lacking for soybean. This stands in contrast to recent work on wheat (*Triticum aestivum* L.) and maize (*Zea mays* L.) grain yield, where comparative analyses of VCU systems across Europe have proven highly informative (Ramakers et al. 2026). That study not only shed light on the genetic progress within countries but also highlighted the potential of rethinking current MET configurations, whether through restructuring national networks or integrating efforts across borders, to improve the efficiency and impact of variety evaluation. For soybean, a similar appraisal is needed.

In this study, we report genetic trends and VCU-network precision in soybean grain yield, protein yield, and protein content in Austria and France. Using rigorous mixed-model methodology, we analysed (1) genotypic and GEI variation, (2) genetic gain over time, and (3) the efficiency of current and alternative trialling systems for grain yield. For both countries, unique historical VCU data were compiled and analysed as part of the EU-H2020 project INVITE. We propose—and illustrate—improving VCU testing in both countries by extending the catalogue of varieties to be compared, simply by merging historical data.

## Methods

### Data

**Fig. 1:**
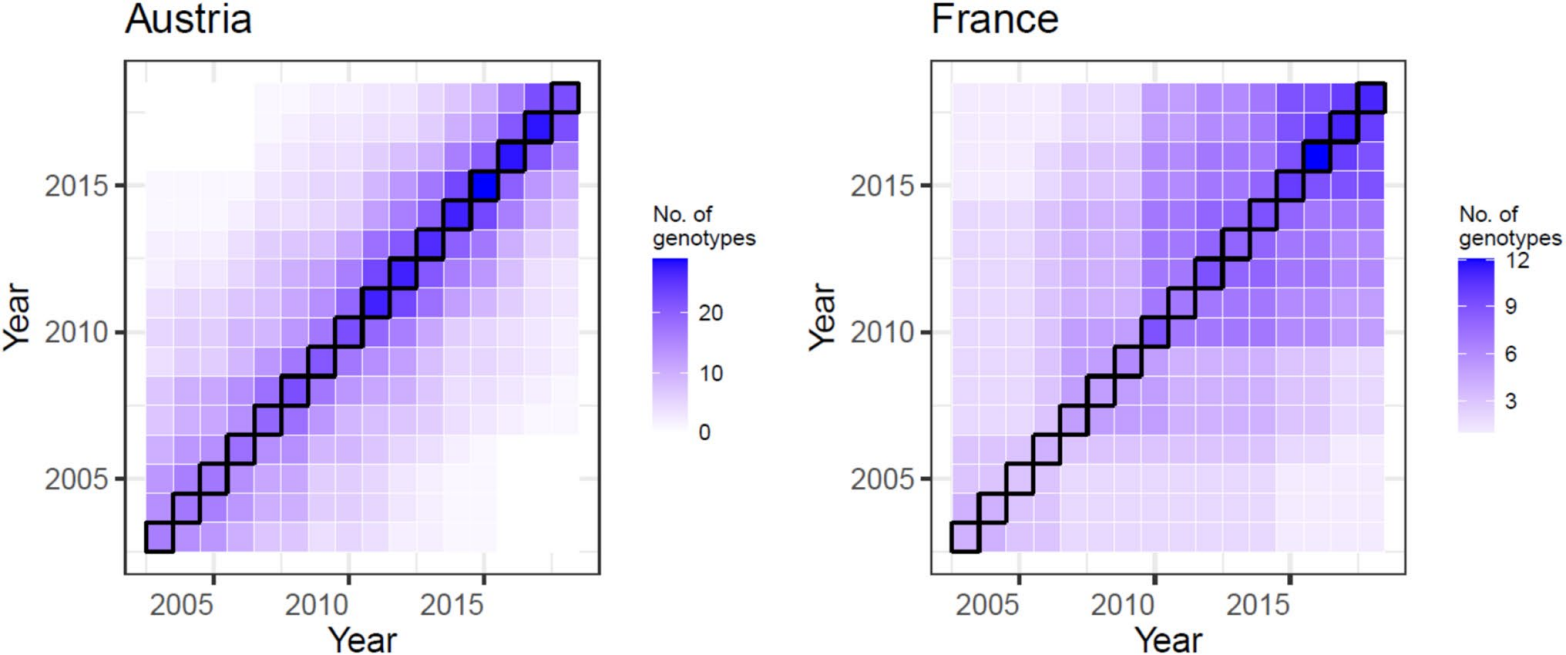
Number of unique soybean varieties per year (diagonal) and the number of varieties common between years (off-diagonal) in Austria and France. Note the different colour scaling between the countries

Phenotypic soybean data were obtained for Austria (AT) via the Austrian Agency for Health and Food Safety (AGES; https://www.ages.at) and for France (FR) via the French Variety and Seed Study and Control Group (GEVES; https://www.geves.fr), for the years spanning 2003–2018 (Table 1). Traits considered were grain (or seed) yield adjusted to 0% moisture (t ha^−1^), protein yield (t ha^−1^) and protein content relative to dry-weight yield (%). Data were available for 5–12 (median 8) test locations per year in AT and 4–22 (median 10) locations in FR, testing a total of 81 and 20 varieties (originally 21) across 2 and 4 maturity groups, respectively, with an overlap of 15 varieties between countries. Due to difficulties in accessing data, the number of varieties in FR was limited, possibly impacting our conclusions regarding genetic trends and testing precision. Additionally, in FR, one variety (out of 21 total) had an extremely early registration date (1991) impacting the genetic trends; this variety was removed from the dataset (present in 10 trials in 2003 and 2004; see below). The total number of location × year combinations was 131 for AT and 177 for FR; maturity groups were tested in different (sub) trials within a location, rendering the actual number of trials higher (Table 1). Both countries had a typical test-cycle length of two years, but the dataset contained standard varieties tested in multiple years. In FR, data included post-registration varieties tested for one additional year. The median overlap of varieties between pairs of years was 6 (AT) and 3 (FR; Fig. 1). Phenotypes were provided at the level of the variety by trial (i.e. variety means adjusted for the experimental design).

**Table 1:** Description of the soybean datasets analysed in this study

Country	No. of years	No. of locations	No. of loc × year	No. of loc × year × maturity	Maturity groups^†^	Median no. of locations per year (range)	Typical no. of test years p. variety	No. of varieties
Austria	16	18	131	197	Early	8 (5–12)	2	81
					Medium-early			
France	16	73	177	232	Very early	10 (4–22)	2	20^‡^
					Early			
					Semi-early			
					Semi-late/late			

^†^Each country had its own maturity classification system (common cultivars may have different maturity designations)

^‡^One variety was removed due to its very early registration date

To estimate the genetic trend in our traits, we ideally would have the first year of testing (FYOT) or year of registration for each variety. Assuming that all varieties were registered at the end of the second year of testing, we could determine FYOT in FR for 14 out of (originally) 21 varieties; for AT, this information was completely lacking. Since the remaining 7 varieties in FR would need to be inferred from the first year of appearance (FYOA) in the dataset anyway, we opted to use FYOA as a surrogate for FYOT in both datasets to make results comparable. Excluding the variety registered in 1991 in FR, FYOT and FYOA correlated strongly (*n* = 13, *r* = 0.95), whereas this correlation dropped substantially when including this variety (*n* = 14, *r* = 0.82). Removing this old variety from further analysis ensured that the genetic trend was (1) less biased and (2) reflective of the relevant study period (2003–2018).

### Modelling G × E in grain yield, protein yield and protein content

Throughout, grain yield and protein yield were analysed in their original scale (t ha^−1^); protein content (%) was logit-transformed prior to analysis ($\ln (p/ \left( {1 - p} \right)$), where *p* = [protein content]/100.

The mixed model for analysis of MET data, using genotypic means, is

1 $$\begin{aligned} z_{ijkm} = & \mu + L_{j} + \gamma t_{k} + Y_{k} + LY_{jk} + S\left( {LY} \right)_{{l\left( {jk} \right)}} + M_{m} + G_{i\left( m \right)} \\ & + \beta r_{i} + GL_{i\left( m \right)j} + GY_{i\left( m \right)k} + ML_{mj} + MY_{mk} \\ & + MLY_{mjk} + GLY_{i\left( m \right)jk}{\prime} \\ \end{aligned}$$

In (1), $z_{ijkm}$ is the mean trait value of the *i*th variety in the *j*th location and the *k*th year from the *m*th maturity group $(i = 1, \ldots ,n_{g} ;j = 1, \ldots ,n_{l} ;k = 1, \ldots , n_{y} ;m = 1, \ldots ,n_{m}$) and μ is the overall mean. Environmental effects included $L_{j}$, the main effect of the *j*th location, $Y_{k}$, the main effect of the *k*th year, $LY_{jk}$, the interaction effect of the *j*th location and *k*th year, $S\left( {LY} \right)_{{l\left( {jk} \right)}}$ (in FR only), the effect of the *l*th subtrial nested within *jk*th trial and a trial-year effect to capture a non-genetic, agronomic or climate trend ($\gamma$), with $t_{k}$ being the discrete trial year scaled to the first year (2003 = 0). Genotypic effects included $M_{m}$, the main effect of the *m*th maturity group, $G_{i\left( m \right)}$, the main effect of the *i*th variety nested within maturity group *m*, $\beta r_{i}$, a genotypic effect to capture the genetic trend ($\beta$), with $r_{i}$ being the discrete FYOA for the *i*th variety (2003 = 0), $GL_{i\left( m \right)j}$, the interaction effect of the *i*th genotype and *j*th location; $GY_{i\left( m \right)k}$, the interaction effect of the *i*th genotype and *k*th year, and $ML_{mj}$, $MY_{mk}$ and $MLY_{mjk}$, the maturity group-by-location, -year, and -location-by-year interaction, respectively. In FR, maturity groups were fully confounded with sub-trials within location-by-year and therefore the two-way interactions ML and MY were omitted. Due to the lack of plot-level data, the three-way genotype-location-year interaction (*GLY*) and the plot error ($\varepsilon_{i(m)jk}$) were confounded and were represented collectively in the term $GLY_{i\left( m \right)jk}{\prime}$. While this compounded term is necessitated by the data, we note that it limits the biological interpretation of higher-order interactions, although it should not substantially compromise the estimation of trialling precision (see “Evaluating the precision of current and alternative VCU systems”). Plot-error variance enters the variance of $GLY_{i\left( m \right)jk}{\prime}$ as the variance of a variety mean, so divided by the number of replicates; hence, $GLY_{i\left( m \right)jk}{\prime}$ is mainly comprised of the three-way interaction effect. Although *GLY* variance can be separated from the plot-error variance using information regarding the precision of each trial (error mean squares, coefficients of variation, etc.), we did not have this information for each trait for both countries, and we therefore adopted a two-stage, unweighted modelling approach—as outlined by Damesa et al. (2017)—throughout. All random terms in model (1) were assumed to follow a normal distribution, i.e. $L_{j} \sim N\left( {0,\sigma_{L}^{2} } \right)$, $Y_{k} \sim N\left( {0,\sigma_{Y}^{2} } \right)$, $LY_{jk} \sim N\left( {0,\sigma_{LY}^{2} } \right)$, $S\left( {LY} \right)_{{l\left( {jk} \right)}} \sim N\left( {0,\sigma_{{S\left( {LY} \right)}}^{2} } \right)$, $G_{i\left( m \right)} \sim N\left( {0,\sigma_{G}^{2} } \right)$,$GL_{i\left( m \right)j} \sim N\left( {0,\sigma_{GL}^{2} } \right)$, $GY_{i\left( m \right)k} \sim N\left( {0,\sigma_{GY}^{2} } \right)$, $ML_{mj} \sim N\left( {0,\sigma_{ML}^{2} } \right)$, $MY_{mk} \sim N\left( {0,\sigma_{MY}^{2} } \right)$, $MLY_{mjk} \sim N\left( {0,\sigma_{MLY}^{2} } \right)$, and $GLY_{i\left( m \right)jk}{\prime} \sim N\left( {0,\sigma_{GLY^{\prime}}^{2} } \right)$.

Given the unbalanced nature of the MET data, cross-network heritability of the traits was reported as Cullis et al.’s (2006) generalized heritability,

2 $$H_{{{\mathrm{Cullis}}}}^{2} = 1 - \left( {\frac{{\overline{v}_{\Delta ..}^{BLUP} }}{{2\hat{\sigma}_{G}^{2} }}} \right)$$

where $\overline{v}_{{{\Delta }..}}^{{{\mathrm{BLUP}}}}$ is the average variance of a difference between predictions of two varieties (BLUPs, i.e. assuming a random genotypic effect). This version reduces to the standard (plot-level) heritability in balanced settings (Schmidt et al. 2019), but is more appropriate for our data as it explicitly accounts for differences in BLUP accuracies between varieties. $H_{{{\mathrm{Cullis}}}}^{2}$ reflects uncertainty in variety ranking induced by BLUP shrinkage, providing a more relevant measure of the reliability of genotype comparisons in our data (see Schmidt et al. 2019 for further discussion of its properties and advantages).

Models were implemented in Asreml-R v. 4 (Butler et al. 2017).

### Modelling genotypic correlations between traits

To estimate the genotypic correlation between two traits, model (1) was expanded to the bivariate form—rather than the trivariate form—to keep the model computationally feasible. For each pair of traits, disregarding the $S_{{l\left( {jk} \right)q}}$ effect for AT, the model was

3 $$\begin{aligned} z_{ijkmq} = & \mu_{q} + L_{jq} + \gamma_{q} t_{kq} + Y_{kq} + LY_{jkq} + S\left( {LY} \right)_{{l\left( {jk} \right)q}} + M_{mq} \\ & + G_{i\left( m \right)q} + \beta_{q} r_{iq} + GL_{i\left( m \right)jq} + GY_{i\left( m \right)kq} \\ & + MLY_{mjkq} + GLY_{i\left( m \right)jkq}{\prime} \\ \end{aligned}$$

In (3), model terms were largely as before, with *q* denoting the *q*th trait (*q* = 1, 2); $ML_{mj}$ and $MY_{mk}$ were dropped since they produced zero-bounded estimates. Independent variances for each trait were estimated for the *L*, *Y*, *LY*,* S,* and *MLY* effect. The remaining random terms, i.e. *G*, *GL*, *GY*, and *GLY’*, were modelled using an unstructured variance–covariance matrix between traits 1 and 2, i.e.

$$\begin{gathered} \left[ {\begin{array}{*{20}c} {G_{1} } \\ {G_{2} } \\ \end{array} } \right]_{i\left( m \right)} \sim N\left( {\left[ {\begin{array}{*{20}c} 0 \\ 0 \\ \end{array} } \right], \left[ {\begin{array}{*{20}c} {\sigma_{G1}^{2} } & {\rho_{G} \sigma_{G1} \sigma_{G2} } \\ {\rho_{G} \sigma_{G2} \sigma_{G1} } & {\sigma_{G2}^{2} } \\ \end{array} } \right]} \right), \hfill \\ \left[ {\begin{array}{*{20}c} {GL_{1} } \\ {GL_{2} } \\ \end{array} } \right]_{i\left( m \right)j} \sim N\left( {\left[ {\begin{array}{*{20}c} 0 \\ 0 \\ \end{array} } \right], \left[ {\begin{array}{*{20}c} {\sigma_{GL1}^{2} } & {\rho_{GL} \sigma_{GL1} \sigma_{GL2} } \\ {\rho_{GL} \sigma_{GL2} \sigma_{GL1} } & {\sigma_{GL2}^{2} } \\ \end{array} } \right]} \right), \hfill \\ \left[ {\begin{array}{*{20}c} {GY_{1} } \\ {GY_{2} } \\ \end{array} } \right]_{i\left( m \right)k} \sim N\left( {\left[ {\begin{array}{*{20}c} 0 \\ 0 \\ \end{array} } \right], \left[ {\begin{array}{*{20}c} {\sigma_{GY1}^{2} } & {\rho_{GY} \sigma_{GY1} \sigma_{GY2} } \\ {\rho_{GY} \sigma_{GY2} \sigma_{GY1} } & {\sigma_{GY2}^{2} } \\ \end{array} } \right]} \right),{\mathrm{and}} \hfill \\ \left[ {\begin{array}{*{20}c} {GLY_{1}{\prime} } \\ {GLY_{2}{\prime} } \\ \end{array} } \right]_{i\left( m \right)jk} \sim N\left( {\left[ {\begin{array}{*{20}c} 0 \\ 0 \\ \end{array} } \right], \left[ {\begin{array}{*{20}c} {\sigma_{{GLY_{1}{\prime} }}^{2} } & {\rho_{{GLY{\prime} }} \sigma_{{GLY_{1}{\prime} }} \sigma_{{GLY_{2}{\prime} }} } \\ {\rho_{{GLY{\prime} }} \sigma_{{GLY_{2}{\prime} }} \sigma_{{GLY_{1}{\prime} }} } & {\sigma_{{GLY_{2}{\prime} }}^{2} } \\ \end{array} } \right]} \right) \hfill \\ \end{gathered}$$

where $\rho_{G}$, $\rho_{GL}$, $\rho_{GY}$, and $\rho_{{GLY^{\prime } }}$ represent the correlations between traits.

### Evaluating the precision of current and alternative VCU systems

To assess the precision of the trialling system, we followed the rationale of Ramakers et al. (2026), where we used the variance components related to the *GL*, *GY* and *GLY'* terms to compute Least Significant Differences (*LSD*) between varieties. Our formulation of *LSD* is based on the variance of a difference between means of two varieties *i* and $i\prime$, $\sigma_{{u_{i} - u_{{i^{\prime}}} }}^{2}$, which, assuming balanced data and based on a model fitted to plot-level data (single-stage analysis; Damesa et al. 2017), can be estimated as

4 $$\sigma_{{u_{i} - u_{{i^{\prime}}} }}^{2} = 2\left( {\frac{{\sigma_{GL}^{2} }}{{n_{L} }} + \frac{{\sigma_{GY}^{2} }}{{n_{Y} }} + \frac{{\sigma_{GLY}^{2} }}{{n_{L} n_{Y} }} + \frac{{\sigma_{\varepsilon }^{2} }}{{n_{L} n_{Y} n_{R} }}} \right)$$

where $n_{L}$, $n_{Y}$ and $n_{R}$ are the number of test locations (median 8 and 10 for AT and FR, respectively), the number of test years (2) and the number of replicates, respectively, and where $\sigma_{\varepsilon }^{2}$ is the plot-error variance (Piepho et al. 2022). Since the *GLY* and plot-error effect cannot be separated in our models, the residuals have variance $\sigma_{{GLY{\prime} }}^{2} = \sigma_{GLY}^{2} + \frac{{\sigma_{\varepsilon }^{2} }}{{n_{R} }}$, assuming homogeneity of error variances across trials (location-years). Hence, Eq. (4) reduces to

5 $$\sigma_{{u_{i} - u_{{i^{\prime}}} }}^{2} = 2\left( {\frac{{\sigma_{GL}^{2} }}{{n_{L} }} + \frac{{\sigma_{GY}^{2} }}{{n_{Y} }} + \frac{{\sigma_{{GLY{\prime} }}^{2} }}{{n_{L} n_{Y} }}} \right)$$

Since both $\sigma_{GLY}^{2}$ and $\sigma_{\varepsilon }^{2}$ are scaled by the same factor $n_{L} n_{Y}$ in the denominator, combining them into $\sigma_{{GLY^{\prime } }}^{2}$ yields equivalent formulations of $\sigma_{{u_{i} - u_{{i^{\prime}}} }}^{2}$. To explore the precision of alternative VCU systems, we experimentally varied $n_{L}$ and $n_{Y}$ in (5) from 1 to 50 and 1 to 10, respectively.

Using $\sigma_{{u_{i} - u_{{i^{\prime}}} }}^{2}$, and assuming level of significance $\alpha$, we can compute *LSD* for grain yield and protein yield as

6 $$LSD = t_{\alpha /2} \cdot \sqrt {\sigma_{{u_{i} - u_{{i^{\prime}}} }}^{2} } \cong 2 \cdot \sqrt {\sigma_{{u_{i} - u_{{i^{\prime}}} }}^{2} }$$

where $t_{\alpha /2}$ is the ($1 - \alpha /2$) quantile of the *t*-distribution for the error degrees of freedom at $\alpha = 0.05$ and $\sqrt {\sigma_{{u_{i} - u_{{i^{\prime}}} }}^{2} }$ is the standard error of a difference (SED) between varieties. For protein content, which was analysed on the logit scale, *LSD* was back-transformed to the proportion scale to facilitate its expression on a relative scale (see below), using

7 $$LSD_{inv. \log it} \cong 2 \cdot \sqrt {\sigma_{{u_{i} - u_{{i^{\prime}}} }}^{2} } \cdot \left( {\mu_{p} \left( {1 - \mu_{p} } \right)} \right)$$

where $\mu_{p} \left( {1 - \mu_{p} } \right)$ represents the derivative of the inverse logit function ($\frac{{e^{\mu } }}{{1 + e^{\mu } }}$) and $\mu_{p}$ is the back-transformed intercept from model (1).

*LSD* was expressed as a percentage of the 0.95 quantile (Q_0.95_) of the trait in question to make it more relevant for breeders, i.e.

8 $$LSD_{\mathrm{\%}} \cong 100\frac{LSD}{{Q_{0.95} }}$$

where ${\mathrm{LSD}}_{{{\% }}}$ represents the margin by which new varieties must differ from high-performing standard varieties at the end of the two-year test period to be considered statistically superior or inferior. A lower margin indicates a more precise system.

### Combining VCU networks to improve precision

If networks correlate at the genotypic level, combining them is an effective way of improving precision of pairwise testing of varieties (Piepho & Malik 2025). This means that, without additional testing, varieties can be compared even when they have not been tested within the same network. To do this, we adapted model (1) to

9 $$\begin{aligned} z_{ijkmp} = & \mu_{p} + L_{jp} + \gamma_{p} t_{k} + Y_{kp} + LY_{jkp} + S\left( {LY} \right)_{{l\left( {jk} \right)p}} + M_{mp} \\ & + G_{i\left( m \right)p} + \beta r_{i} + GL_{i\left( m \right)jp} + GY_{i\left( m \right)kp} \\ & + MLY_{mjkp} + GLY_{i\left( m \right)jkp}{\prime} \\ \end{aligned}$$

In (9), which was fitted for each trait separately, elements are largely as in Eq. (3), but now with subscript *p* (rather than *q*) denoting the country (*p* = 1, 2), with a country-specific intercept ($\mu_{p}$), maturity group ($M_{mp}$) and environmental trend ($\gamma_{p} t_{k}$) effect; the genotypic trend ($\beta r_{i}$) was fitted across both countries. All random terms were made country specific, mostly via a diagonal structure to estimate separate variances components per country. For the random genotypic ($G_{i\left( m \right)p}$), year ($Y_{kp}$) and genotype-by-year ($GY_{i\left( m \right)kp}$) effects, an unstructured variance–covariance matrix was assumed to estimate the correlation between countries (Malik et al. 2022).

Based on the current network configurations, we constructed a balanced dataset for the three traits based on ten locations, two test years and two replicates for each country. Based on this balanced dataset, we could use the mixed model, with variance components and correlations fixed to those estimated from model (9), to compute pairwise (variety-to-variety) SEDs (see Piepho & Malik 2025 for details). An alternative model was fitted, in which the genotypic (and year) correlations between countries ($\rho_{G}$, $\rho_{Y}$, and $\rho_{GY}$) were fixed at 0. This mimics the current situation, where varieties are evaluated separately for each country. From this model, *LSD*s were computed based on average, pairwise SEDs, rather than the formulation in Eq. (6). In Eq. (6), SED is based on $\sigma_{{u_{i} - u_{{i^{\prime}}} }}^{2}$ and pertains to the adjusted genotypic means. Here, SEDs are pairwise calculations obtained from the generalized inverse of the coefficient matrix from the mixed model (see Piepho & Malik 2025 for details) and pertain to the random genotypic main effect; the resulting *LSD* values are presented as averages across variety pairs. Due to this distinction—and because the model (1) was based on different (and unbalanced) network configurations—these different formulations of *LSD* are not identical. The interest here, therefore, is in the comparison of *LSD*s computed with or without the inclusion of the genotypic correlation between networks, based on models with random genotypic effects.

*LSD*s (relative to the 0.95 quantile) were averaged across variety pairs within the following comparisons (Piepho & Malik 2025; Ramakers et al. 2026):

- (***i***, ***ii***) Both varieties occur in both networks; *LSD*_%_ is computed for network 1 (*i*) or network 2 (*ii*).
- (***iii***, ***iv***) Both varieties occur only in network 1; *LSD*_%_ is computed for network 1 (*iii*) or network 2 (*iv*).
- (***v***, ***vi***) Both varieties occur only in network 2; *LSD*_%_ is computed for network 1 (*v*) or network 2 (*vi*).
- (***vii***) One variety occurs only in network 1 and the other only in network 2; *LSD*_%_ is computed for network 1.
- (***viii***) One variety occurs only in network 1 and the other only in network 2; *LSD*_%_ is computed for network 2.

When $\rho_{G}$, $\rho_{Y}$ and $\rho_{GY}$ equal zero, comparisons (*i*) and (*iii*), as well as (*ii*) and (*vi*), yield the same results—given the fully balanced design—because no information is shared between networks. When $\rho_{G} ,\rho_{Y} ,\rho_{GY}> 0$, *LSD*_%_ is expected to improve within comparisons (*i*) and (*ii*), as it effectively mimics an increase in the number of test locations; on the contrary, we may expect no improvement within comparisons (*iii*) and (*vi*), since both testing and comparison are done within the same network. An interesting and challenging scenario arises when there are no observations in the focal network for one (*vii*, *viii*) or both varieties (*iv*, *v*). In these cases, leveraging the genotypic correlation is expected to substantially improve precision (Piepho & Malik 2025).

## Results

### Genotypic and genotype-by-environment variation and genotypic correlations between traits

**Fig. 2:**
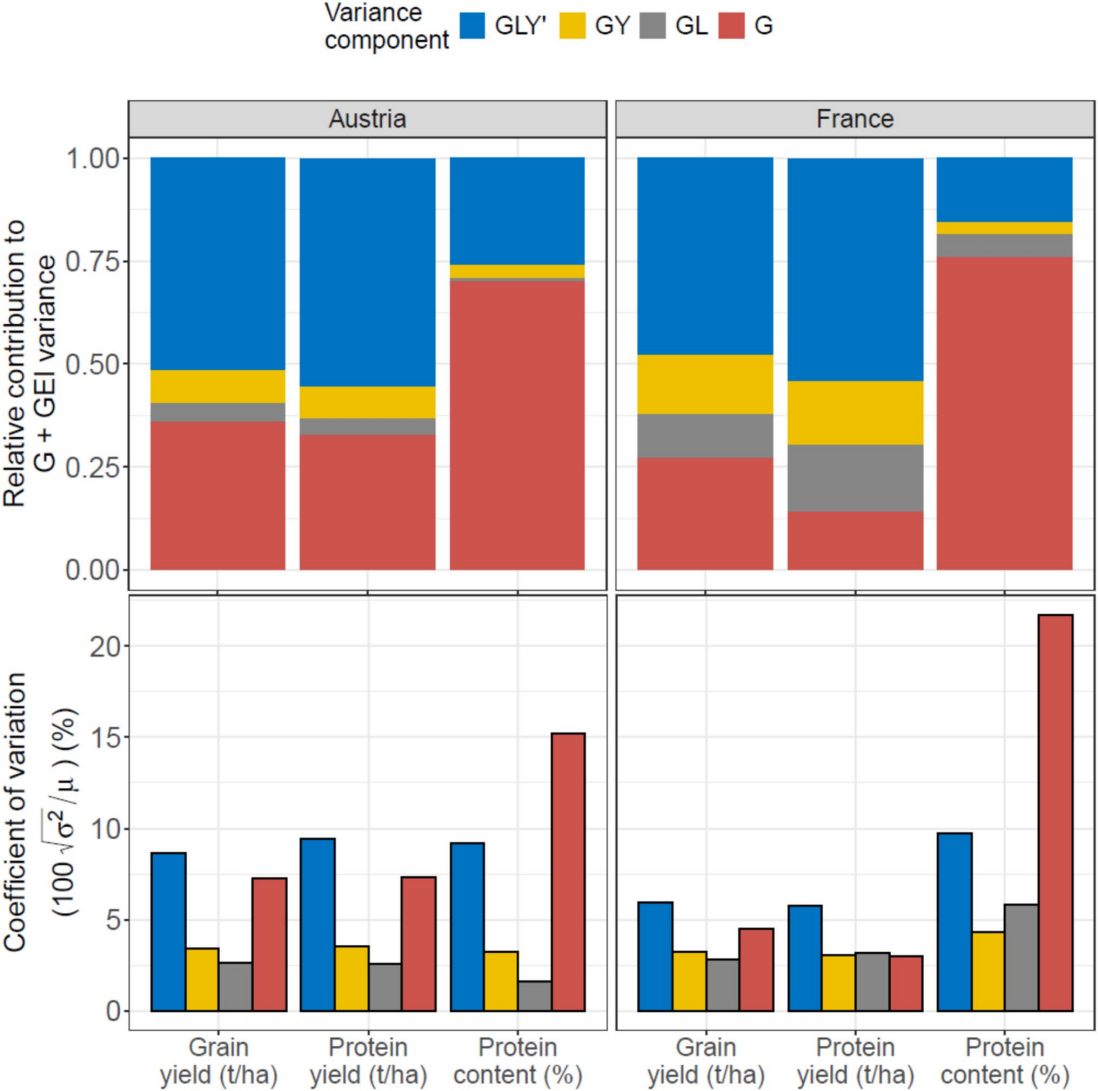
Variance components proportional to the total genetic variance (top) and their coefficients of variation (bottom) for protein content, protein yield and grain yield in Austria and France

Genotypic variation for each trait and country is displayed in Fig. 2 (full results, including generalized *H*^2^, in Supplementary Table S1). Both AT and FR networks exhibited similar partitioning of genotypic variation, with G and *GLY*' being the largest source of variation ranging (14–36% and 48–55%, respectively), and relatively small *GL* and *GY* effects, for both grain yield and protein yield. For protein content, the main *G* effect was much more pronounced, accounting for 70–76% of the genotypic variation. Expressing the variance components as the coefficient of variation (CV, standard deviation over the intercept), the *G*, *GL*, *GY* and *GLY'* components for grain yield and protein yield in the two networks amounted to 2.8–7.3%, 2.5–3.2%, 3.1–3.6% and 5.8–9.4%, respectively. For protein content, CV_G_ (i.e. for the genotypic main effect) was as high as 15.2–21.2%. Assuming that, given normally distributed random effects, 95% of the genotypic values lie within twice the standard deviations from the mean in both directions, this means that the genotypic main effect accounted for ± 5.6% to ± 14.6% around the mean grain yield and protein yield, whereas this was ± 30.4% to ± 42.4% for protein content. Genotypic variation in protein content appeared more stable across environments (i.e. less GEI compared to main *G* variance) than either grain yield or protein yield, in both countries.

In both AT and FR, grain yield and protein yield exhibited a strong genotypic correlation ($\rho_{G}$; Table 2). The same was true for $\rho_{GL}$, $\rho_{GY}$ and $\rho_{{GLY{\prime} }}$, i.e. the relative magnitude of variances for GEI effects was similar between traits. Grain yield exhibited a moderate, negative genotypic correlation with protein content, although only significantly so in AT, whereas protein yield and protein content correlated positively but non-significantly in both countries. These results suggest that grain yield and protein yield could be mutually reinforcing targets of selection, whereas grain yield and protein content are not.

**Table 2:** Genotypic correlations (± SE) between the different traits, at the level of genotype (*G*), genotype-by-location (*GL*), genotype-by-year (*GY*), and the combined three-way interaction + plot-error effect (*GLY*'). The correlations for Austria are given on the upper diagonal, whereas the correlations for France are given on the lower diagonal

		Grain yield (t ha^−1^)	Protein yield (t ha^−1^)	Protein content (logit(*p*))
Grain yield (t ha^−1^)	*G*		*0.838* ± 0.038***	*–0.357* ± 0.110**
	*GL*		0.924 ± 0.034	−0.600 ± 0.357
	*GY*		0.969 ± 0.010	−0.259 ± 0.142
	*GLY'*		0.948 ± 0.003	0.005 ± 0.029
Protein yield (t ha^−1^)	*G*	*0.650* ± 0.204*		*0.201* ± *0.121*^* ns*^
	*GL*	0.948 ± 0.030		−0.367 ± 0.430
	*GY*	0.968 ± 0.013		−0.004 ± 0.151
	*GLY'*	0.971 ± 0.003		0.251 ± 0.027
Protein content (logit *p*))	*G*	*-0.754* ± 0.148**	*0.033* ± *0.340*^* ns*^	
	*GL*	0.504 ± 0.210	0.679 ± 0.153	
	*GY*	-0.179 ± 0.203	0.069 ± 0.209	
	*GLY'*	-0.118 ± 0.060	0.155 ± 0.059	

Note: statistical significance tested for the main genotypic correlation only (in italics), using likelihood-ratio tests with 1 degree of freedom, and with *p* values corrected for multiple testing (Benjamin–Hochberg). *p < 0.05; **p < 0.01; ***p < 0.001; ns = not significant

### Precision of current and alternative VCU networks

**Fig. 3:**
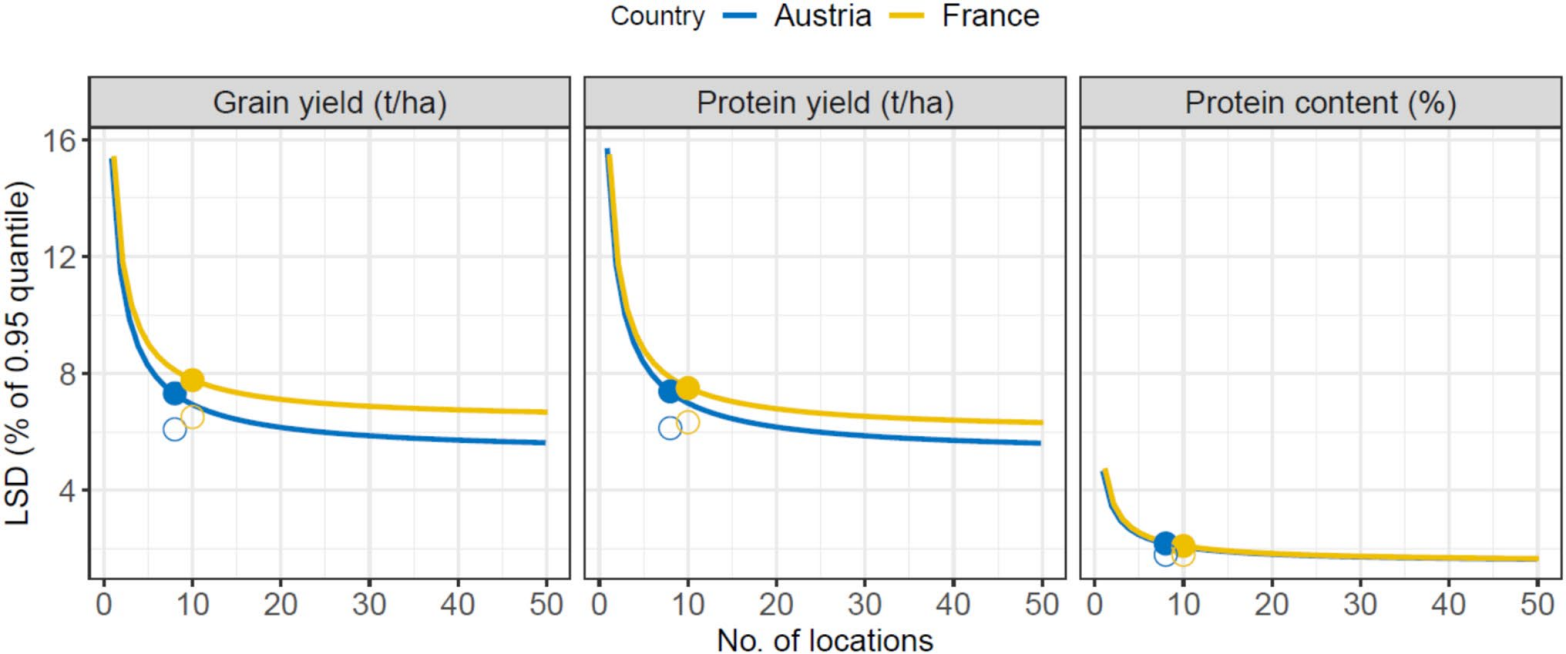
Least Significant Differences (*LSD*) as a function of the number of locations for three traits in Austria and France. *LSD*s are expressed as a percentage of the 0.95 quantile in the trait. The closed circles denote the *LSD*_**%**_ in the current system (i.e. assuming 2 test years and 8 and 10 locations, respectively), whereas the open symbols denote the *LSD*_%_ if the systems adopted 3 test years; the lines are projections across numbers of locations, keeping the number of test years constant at 2

Current network precision, given a typical 2 test years and 8 (AT) or 10 (FR) test locations, resides at 7.3% and 7.8% for grain yield, 7.4% and 7.5% for protein yield, and 2.2% and 2.1% for protein content, respectively (closed symbols in Fig. 3). Thus, current networks can distinguish between top-tier varieties that are ~ 7.5% apart in terms of grain yield and protein yield, and ~ 2% in terms of protein content. The substantially higher precision for protein content is directly mirrored by the strong genotypic main effect relative to G × E effects (Fig. 2). Recalling the total spread in genotypic values (roughly 4 standard deviations, i.e. 11.2–29.2% for grain yield and protein yield and 60.8–84.8% for protein content), the *LSD*_%_ for grain yield and protein yield appear insufficient to distinguish between high-performing (top 5%) varieties, whereas the *LSD*_%_ for protein content may be sufficient for this purpose (i.e. ratio *LSD*_%_/spread is 0.25–0.63 vs. 0.02–0.04, respectively).

Rearranging the VCU networks by changing the number of locations showed that both VCU networks could increase precision by increasing the number of test locations (lines in Fig. 3). However, this effect was marginal, considering the position of the current *LSD*_%_ in the elbow point of the projection curves. A more efficient gain had perhaps be obtained from going from 2 to 3 test years; for the current, typical number of test locations, this results in a reduction of 1.2 percentage points (%pt.) and 1.3%pt. (grain yield), 1.3%pt. and 1.2%pt. (protein yield), and 0.4%pt. and 0.3%pt. (protein content), in AT and FR, respectively (open symbols in Fig. 3).

### Genetic trends in protein content, protein yield and grain yield

**Fig. 4:**
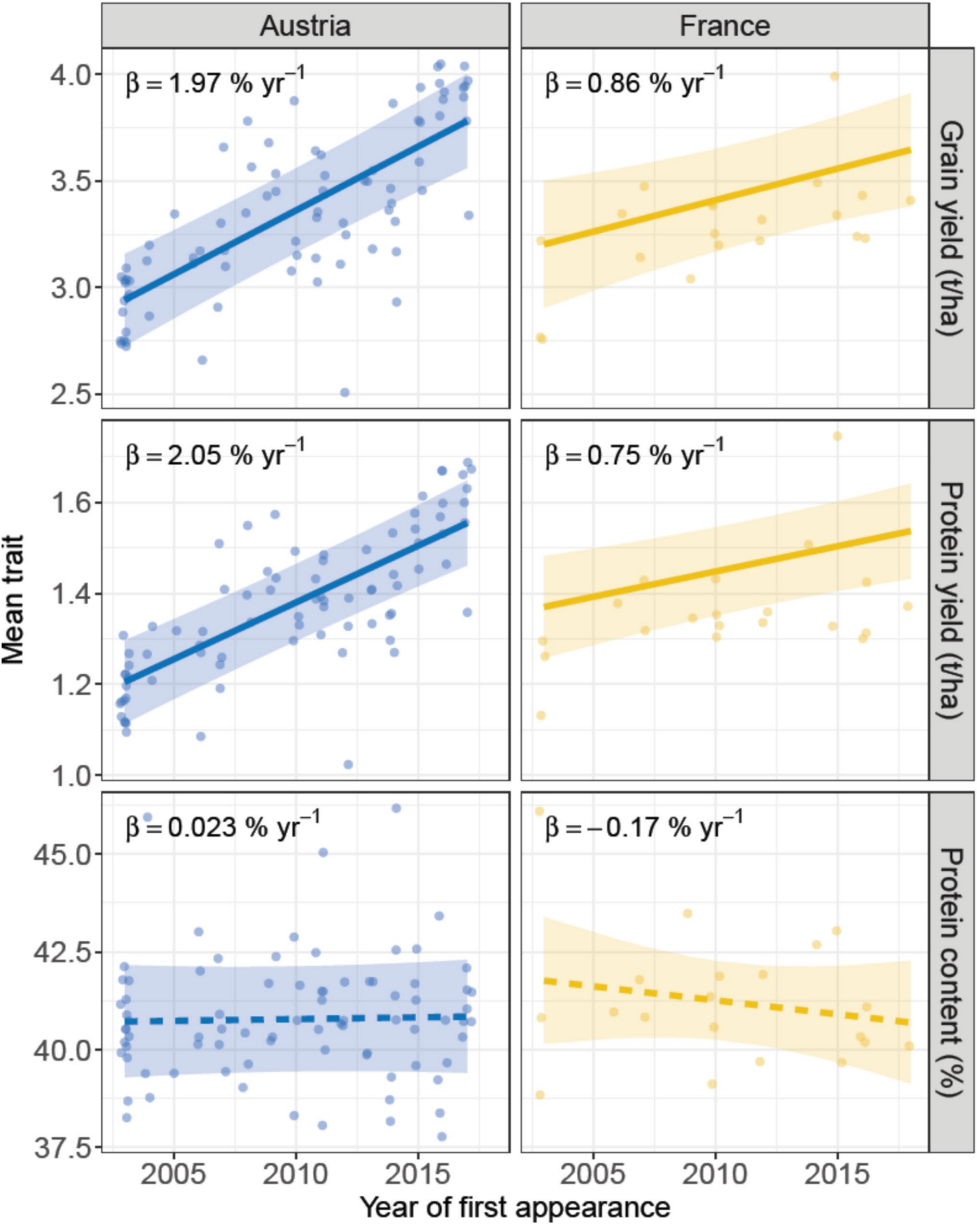
Genetic trends ($\beta$, in % yr^−1^) in soybean grain yield (t ha^−1^), protein yield (t ha^−1^), and protein content (%) in historical VCU-trial networks in Austria and France. Points are variety-specific means (across years and locations; i.e. model (1) with a fixed variety effect) plotted vis-à-vis the first year of testing of a variety (notice some horizontal jittering). Lines and shadings are predictions and 95% CIs for the genetic trend line from the fixed-effect terms in model 1, with solid lines indicating statistical significance (p < 0.05) and dashed lines the lack of statistical significance (p > 0.05). Points and estimates for protein content were back-transformed from the logit scale

Estimated genetic gains within released varieties wwew significantly positive for protein yield and grain yield in both countries, but not protein content (Fig. 4). In the latter trait, the trend was slightly positive (0.02% yr^−1^; AT) or negative (− 0.17% yr^−1^; FR) and non-significant. For protein yield (t ha^−1^), genetic gain amounted to 2.05% yr^−1^ (AT) and 0.75% yr^−1^ (FR), whereas for grain yield, the gain amounted to 1.97% yr^−1^ (AT) and 0.86% yr^−1^ (FR). Non-genetic (agronomic) trends were in all cases non-significant, and all but protein content in AT were negative (see Supplementary Table S1 for detailed estimates).

### Combining VCU networks to improve testing precision

**Fig. 5:**
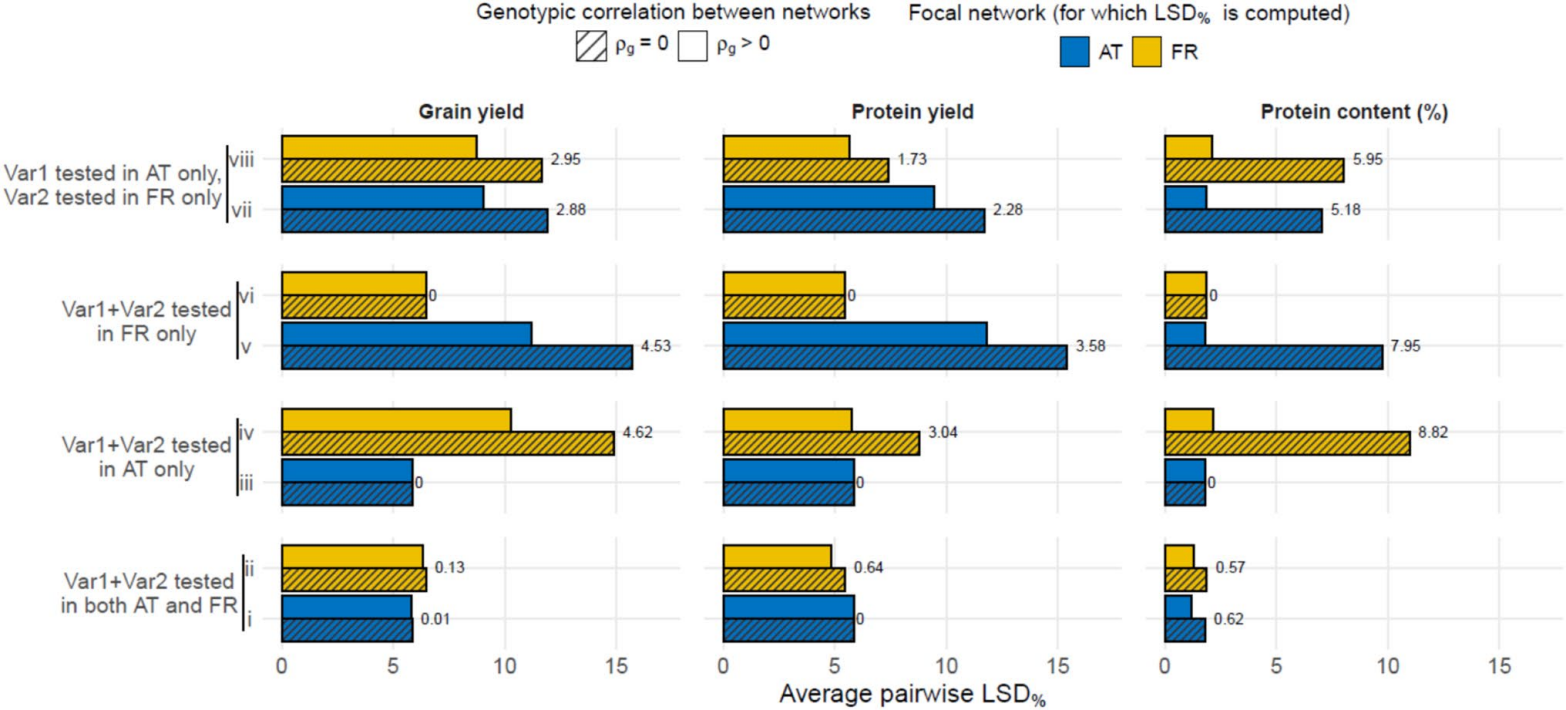
Improvement in average pairwise (variety-to-variety) *LSD*_%_ in grain yield, protein yield, and protein content from combining the Austrian (AT) and French (FR) VCU networks. The horizontal axes show the average pairwise *LSD*_%_ within a focal network (i.e. for which *LSD* is computed), after combining data from AT and FR. The *LSD*_%_ for the focal network is the average of pairwise comparisons between varieties in the following scenarios: (i, ii) both varieties (1 and 2) are tested in both networks; (iii, iv) both varieties are tested only in AT; (v, vi) both varieties are tested only in FR; (vii, viii) one variety is tested in AT and the other in FR. Colours indicate the focal networks. Hatched bars represent no genotypic correlation between networks ($\rho_{G} = 0$), while unhatched bars incorporate the empirically estimated correlation ($\rho_{G}> 0$; see Supplementary Table S2); the difference between bars in percentage points is shown as a number next to them. Note that *LSD*_%_ values reported here are not directly comparable to the *LSD*_%_ values reported elsewhere in the main text (see “Methods” for details)

Combining the two VCU networks allowed us to compute the precision from leveraging the genotypic (and year and genotype–year) correlation between the networks. We were able to estimate the correlations between the networks for each trait (see Supplementary Table S2 for the estimated variance components and correlations), although for protein content the year correlation ($\rho_{Y}$) could not be estimated. Combining networks, assuming a fully balanced dataset with 10 test locations and 2 test years, leveraging the correlation improved network precision by different degrees, depending on where varieties were tested and on the focal network (Fig. 5). Comparisons *i* and *ii*, as well as *iii* and *vi*, are roughly equivalent to the current situation where comparison between varieties is done within the same network (via Eq. 6)—yet *LSD*_%_ values are not identical since the (theoretically) combined network configuration is completely balanced with respect to the number of locations and years.

When both varieties of a pair were tested in both networks (comparisons *i* and *ii*), leveraging the correlation improved *LSD*_%_ slightly (0.001–0.64%pts.) for each trait in both networks, compared to when the correlations were not incorporated (i.e. $\rho_{G} ,\rho_{Y} ,\rho_{GY} = 0$). When both varieties in a pair were tested either in AT only or in FR only (*iv, v*), precision went up by 3.04–8.81% pts. in the other network (compared to when $\rho_{G} ,\rho_{Y} ,\rho_{GY} = 0$). Finally, when one variety in a pair was tested in one country and the other variety in the other (*vii*, *viii*), precision in the focal country went up by 1.73–5.95%pts (Fig. 5).

## Discussion

We report significant genetic trends for grain yield and protein yield, but not protein content, in soybean from registered varieties in Austrian and French VCU networks. Genetic trends in grain yield and protein yield were very similar within networks, in line with the very similar estimates for genotype (*G*) and G × E variations in both traits and the fairly strong genotypic correlation between them ($\rho_{G}$ ≥ 0.650). The relative annual gain was high in AT (1.97–2.05% yr^−1^), not only compared to FR but also compared to previously estimated trends in grain yield for wheat (*Triticum aestivum*; average 0.84% yr^−1^) and maize (*Zea mays*; average 1.13% yr^−1^) in different European VCU systems (Ramakers et al. 2026) (but see discussion below on comparing with different crops). Estimates were, however, within the range of previously reported gains in soy grain and protein yield from different studies (Table 3), albeit at the higher end for AT. The genetic trend in protein content was non-significant in both countries, and negative in the case of FR. The negative direction of the trend aligns with the general observation across studies that protein content has gone down in the past decades, due to its negative genetic correlation with grain yield (Table 2; de Felipe et al. 2016; Milioli et al. 2022; Morrison et al. 2000). The negative trend in protein content in FR seemingly contrasts unpublished results by GEVES, who found an annual increase of 0.21% (A. Gouleau, pers. comm.), but it is uncertain whether this increase was significantly different from 0—and thus from our own negative, non-significant trend.

**Table 3:** Genetic trends in soy grain (seed) yield, protein yield and protein content reported in different studies, for different localities and time periods

Trait (unit)	Reference	Location	Years	Genetic trend (units yr^−1^)^†^	Genetic trend (% yr^−1^)^†‡^
Grain yield (t ha^−1^)					
	de Borja Reis et al. (2020)	KS (US)	1980–2014	0.04	1.48
	De Bruin and Pedersen (2008)	IA (US)	1938–2004	0.025	~ 0.6
	de Felipe et al. (2016)	Argentina	1982–2015	0.034–0.044	0.7–1.1
	Jin et al. (2010)	N-E China	1951–2006	0.01	0.59
	Keep et al. (2016)	KS (US)	1923–2008	0.19–0.35	1.83–3.19
	Milioli et al. (2022)	Brazil	1966–2011	0.014–0.015	0.39–0.42
	Morrison et al. (2000)	ON (Canada)	1934–1992	0.01	0.45
	Rogers et al. (2015)	AR (US)	1928–2008	0.011–0.024	0.41–0.92
	Tamagno et al. (2020)	KS (US)	1980–2013	0.033	0.74
	Todeschini et al. (2019)	Brazil	1996–2011	0.04	2.46
	Weidenbenner et al. (2014)	IL, IN, MN, WI (US)	1928–2008	0.021–0.023	0.81–1.20
Protein yield (t ha^−1^)	de Felipe et al. (2016)	Argentina	1982–2015	0.007–0.011	0.5–1.0
	Tamagno et al. (2022)	KS (US)	1980–2013	0.011	1.14
	Wilson et al. (2014)	IL, IN, MN, WI (US)	1923–2008	0.006–0.010	0.73–1.14
Protein content (g kg^−1^)	de Borja Reis et al. (2020)	KS (US)	1980–2014	−0.122	−0.03
	de Felipe et al. (2016)	Argentina	1982–2015	−0.28 – -0.25	−0.07 – -0.06
	Milioli et al. (2022)	Brazil	1966–2011	−0.024 – -0.021	−0.07– -0.06
	Morrison et al. (2000)	ON (Canada)	1934–1992	−0.537	−0.13
	Rogers et al. (2015)	AR (US)	1928–2008	−0.35 – 0.18	−0.11 – 0.05
	Tamagno et al. (2022)	KS (US)	1980–2013	−0.6	−0.18
	Weidenbenner et al. (2014)	IL, IN, MN, WI (US)	1928–2008	−0.22	−0.06

^†^Ranges are given across different maturity groups, time periods, or geographical regions

^‡^Percent increase was computed relative to the first year in the series

Our estimated genetic trends were similar to those previously reported (Table 3) in that they were all based on registered varieties, but differed in the way they were estimated. Firstly, we did not have registration dates for each variety and thus based our trends on FYOA. This should not bias the trends as long as all varieties in the dataset are candidate varieties, but it will introduce bias when it contains reference varieties registered before the start of the studied period. For FR, we had registration dates for 14 varieties (including an old variety registered in 1991); if we use these registration years in model (1) and assume the registration years for the remaining 7 varieties based on FYOT, the genetic trends increase, notably for grain yield (from 0.030 to 0.042 t ha^−1^, or from 0.86 to 1.33% yr^−1^, assuming 2003 as the reference year) and for protein yield (from 0.011 to 0.017 t ha^−1^, or from 0.75 to 1.16% yr^−1^). This bias is almost entirely removed when we exclude the variety registered in 1991, and in doing so we notice that the trends are now much closer to those based on FYOA only (0.98 vs 0.86% yr^−1^ for grain yield; 0.70 vs 0.75% yr^−1^ for protein yield).

The second way in which our trends differ from those reported in Table 3 is that our estimates were based on historical data (16 trial years and typically 8–10 locations per year), corrected for environmental and long-term G × E effects. In contrast, the reported studies used ‘era’ trials, where a few trials were used, typically over 2 or 3 years and sometimes on the same location (e.g. Morrison et al. 2000), consisting of registered varieties from different eras. A mixed model was typically used to compute genotype effects (e.g. BLUPs), which were subsequently regressed on the release year. The latter approach is logistically convenient as it requires only a few years of testing. When long-term VCU-MET data are already available, it is convenient—and to some extent preferable—to use these data instead, but the interpretation of the estimated genetic trend is likely different. Analysis on long-term MET data, in a diverse set of locations, likely captures environmental and G × E effects more accurately, which leads to a more accurate estimation of genotypic main variance and, potentially, the genotypic trend across time. A caveat, however, is that variety-ageing effects (Andrade et al. 2026; Piepho et al. 2014) are not captured in the trend from long-term data (as varieties leave the system within a few years), whereas they may be captured well for the older varieties in era trials, leading to better estimation of the ‘true’ (i.e. incorporating ageing) genetic trend. Despite being within realistic ranges, therefore, our estimated trends from long-term data may be biased upwards (see discussion in Piepho et al. 2014).

Grain yield and protein yield were highly correlated at the level of the main genotypic and the G × E effects in both networks. In both AT and FR, the absolute genetic trend in grain yield was 2.4–2.7 times that of protein yield (Supplementary Table S1), despite protein content (% or g kg^−1^) remaining stable. This should not be unexpected given that the genotypic correlations are less than unity, and sustained selection on grain yield should lead to a lower correlated response in protein yield (Falconer & Mackay 1996) (note that heritability for both traits is rather similar within each country; Supplementary Table S1). Given the very high heritability of protein content compared to the other traits, there is great scope for improvement in the former by shifting selection towards protein content. This will, however, come at the expense of grain yield—and consequently farmer income—and is therefore arguably an infeasible breeding strategy.

We found low testing precision for grain and protein yield in the AT and FR VCU networks (*LSD*_%_ = 7.3–7.8%), compared to wheat (3.1–7.5%) and maize (3.0–8.2%) grain yield in similar European systems (Ramakers et al. 2026), and in particular to soy protein content in this study (2.1–2.2%). Given the CV_G_, current precision is insufficient to reliably distinguish the top 5% of varieties for yield traits, echoing conclusions for wheat and maize by Ramakers et al. (2026). However, it is possible that part of the lower precision observed in soybean arises from crop biology rather than the VCU system itself. Unlike cereals, which rely on mineral nitrogen uptake, soybean depends on a self-regulated and environmentally sensitive *N*₂-fixation process (e.g. Holland et al. 2023), which may contribute to larger G × E effects in grain yield. Although precision is rather low, it appears sufficient to enable ample genetic gain in grain and protein yield. Assuming our variance estimates reflect early breeding stages, precision in a hypothetical early-stage trial (one location, one year, two replicates), based on eqs. (5, 6), would be 16.2% (AT) and 16.7% (FR) for grain yield. With a ten-year variety development cycle, expected genetic gains would hence be 1.62% yr⁻^1^ in AT (vs. 1.97% yr⁻^1^ observed) and 1.67% yr⁻^1^ in FR (vs. 0.86% yr⁻^1^ observed). This suggests near-optimal selection efficiency in AT, but possible trade-offs with other traits or other limiting factors in FR. Notably, FR tested fewer varieties than AT (or rather, the genetic trend was based on fewer varieties), and the impact of this on the genetic trends remains uncertain.

How can we improve the Austrian and French soybean VCU networks? There are several ways to go about this (see Ramakers et al. 2026), for example, by extending testing to three years to increase precision. Although this option is not always on the table for obvious financial reasons, FR is already including registered varieties in a third round of post-registration testing, effectively increasing connectivity between years. The most straightforward and cost-effective way of improving precision is to combine data from both countries (Fig. 5; Piepho & Malik 2025). When varieties are tested in both networks, combining data offers only a modest gain in precision compared to analysing the networks separately. However, for comparing varieties not jointly tested—a common scenario in European VCU systems—data integration is a cost-effective solution. Importantly, in the most challenging case where both varieties are tested only in one network but comparison is needed in another (*vii–viii* in Fig. 5), precision was comparable to scenarios where one variety is observed in the target network (*iv–v*), particularly for FR, which had most to gain from merging data. This demonstrates that variety comparisons can extend beyond the borders of a network, simply by leveraging the strong correlation between countries (Malik et al. 2022; Piepho & Malik 2025). This correlation can be further leveraged by including multiple shared check varieties across networks and by incorporating genetic marker information to facilitate enhanced genotypic connectivity between networks (Yang et al. 2024).

A key assumption throughout this study has been that the test environments can be regarded as samples from the same distribution. This has allowed the comparison and integration of the two networks but may not accurately reflect biological reality. Environments may experience different climatic conditions, have different soil types, be in different longitudes/latitudes and different elevations, or be subject to different management regimes (as is the case for some wheat and maize VCU networks in Europe; see Ramakers et al. 2026). These factors may influence estimates of G and GEI variances, genetic trends, and testing precision. A more detailed environment characterization (envirotyping) could provide additional insight into differences between networks and help define how VCU systems may be optimized beyond statistical precision alone. Such harmonized environmental data were, however, not available for the present study. For AT, we have trial coordinates and elevation, which suggest some spatial pattern (i.e. higher elevation at trials in southern and western regions), but these variables are insufficient for a robust environmental characterization. Detailed envirotyping efforts would be required to meaningfully characterize environmental heterogeneity, as has recently been done within H2020 INVITE for European wheat networks (Chen et al., submitted). Besides improved interpretation, such envirotyping efforts may generate environmental covariates that reduce residual GEI variation (Araújo et al. 2024; Baptistella et al. 2025; Elmerich et al. 2023; Krause et al. 2025; Welcker et al. 2022) and hence increase precision. These environmental covariates, in turn, could be used to extend genomic prediction models and better estimate the correlations between the VCU networks (Melsen et al. 2025; Piepho & Blancon 2023). Such approaches are currently lacking for the Austrian and French soybean network, but could be a promising future avenue for network optimization.

## Competing interests

The authors declare no competing interests.

## Supplementary Information

Below is the link to the electronic supplementary material.

Supplementary file1 (DOCX 26 KB)

## Data Availability

Data used in this paper are available through Zenodo (https://zenodo.org/records/18024082). R code used in this paper is very similar to that associated with the wheat and maize paper by Ramakers et al. (2026) and can be found with the supplementary material therein.
