# Supplementary material for: Evaluation and improvement of two European soybean VCU networks
Source: Theor Appl Genet. 2026 May 25;139(6):162. doi: 10.1007/s00122-026-05282-x (PMC13199210; doi:10.1007/s00122-026-05282-x)
Supplement: Supplementary file 1 — Supplementary file1 (DOCX 26 KB) [file 122_2026_5282_MOESM1_ESM.docx]

**Supplementary material to ‘Evaluation and optimization of two European soybean VCU networks’**

Jip J.C. Ramakers, Waqas A. Malik, Klemens Mechtler, Christine Fintz, Hans-Peter Piepho, Fred A. van Eeuwijk

*Table S1. Variance components and fixed-effects coefficients (± SE) from the mixed models pertaining to grain yield, protein yield, and protein content (in logit scale).*

| Model term | Austria | | | |  | France | | | |
| --- | --- | --- | --- | --- | --- | --- | --- | --- | --- |
|  | Protein content | | Protein yield | Grain yield |  | Protein content | Protein yield | Grain yield | |
| **Fixed effects** | | *logit(p)* | *t ha^-1^* | *t ha^-1^* |  | *logit(p)* | *t ha^-1^* | *t ha^-1^* | |
| Intercept ($\mu$) ^†^ | -0.4194 ± 0.0379 | | 1.2192 ± 0.0777 | 3.0398 ± 0.1798 |  | -0.3151 ± 0.0379 | 1.4847 ± 0.0840 | 3.4562 ± 0.2064 | |
| Genetic trend ($\beta$) | 0.0004 ± 0.0016^ns^ | | 0.0249 ± 0.0027*** | 0.0599 ± 0.0066*** |  | -0.0029 ± 0.0035 | 0.0111 ± 0.0033** | 0.0296 ± 0.0102** | |
| In % yr^-1 ‡^ | 0.02 | | 2.05 | 1.97 |  | -0.17 | 0.75 | 0.86 | |
| Non-gen. tend ($\gamma$) | 0.0052 ± 0.0028^ns^ | | -0.0017 ± 0.0083^ns^ | -0.0118 ± 0.0191^ns^ |  | -0.0019 ± 0.0024^ns^ | -0.0125 ± 0.0065^ns^ | -0.0276 ± 0.0151^ns^ | |
|  |  | |  |  |  |  |  |  | |
| **Variance components**^§^ | *logit(p)^2^* | | *t^2^ ha^-2^* | *t^2^ ha^-2^* |  | *logit(p)^2^* | *t^2^ ha^-2^* | *t^2^ ha^-2^* |  |
| *Genetic terms* | | |  |  |  |  |  |  | |
| G | 0.0041 ± 0.0007 | | 0.0079 ± 0.0015 | 0.0487 ± 0.0092 |  | 0.0047 ± 0.0016 | 0.0019 ± 0.0011 | 0.0241 ± 0.0116 | |
| GL | 0.0000 ± 0.0000 | | 0.0010 ± 0.0003 | 0.0062 ± 0.0018 |  | 0.0003 ± 0.0001 | 0.0022 ± 0.0006 | 0.0095 ± 0.0033 | |
| GY | 0.0002 ± 0.0000 | | 0.0019 ± 0.0004 | 0.0108 ± 0.0021 |  | 0.0002 ± 0.0001 | 0.0021 ± 0.0006 | 0.0126 ± 0.0034 | |
| GLY' | 0.0015 ± 0.0001 | | 0.0132 ± 0.0005 | 0.0692 ± 0.0028 |  | 0.0009 ± 0.0001 | 0.0073 ± 0.0007 | 0.0421 ± 0.0037 | |
| ML | 0 | | 0 | 0 |  |  |  |  | |
| MY | 0 | | 0 | 0 |  |  |  |  | |
| MLY | 0.0015 ± 0.0003 | | 0.0086 ± 0.0018 | 0.0454 ± 0.0095 |  | 0.0002 ± 0.0001 | 0.0007 ± 0.0011 | 0.0020 ± 0.0061 | |
| *Non-genetic terms* | | |  |  |  |  |  |  | |
| L | 0.0098 ± 0.0045 | | 0.0034 ± 0.0060 | 0.0234 ± 0.0289 |  | 0.0057 ± 0.0014 | 0.0547 ± 0.0151 | 0.2743 ± 0.0751 | |
| Y | 0.0000 ± 0.0000 | | 0.0105 ± 0.0088 | 0.0638 ± 0.0455 |  | 0.0009 ± 0.0006 | 0.0032 ± 0.0038 | 0.0243 ± 0.0217 | |
| LY | 0.0155 ± 0.0022 | | 0.0936 ± 0.0141 | 0.4118 ± 0.0630 |  | 0.0034 ± 0.0006 | 0.0409 ± 0.0073 | 0.1824 ± 0.0340 | |
| S(LY) |  | |  |  |  | 0.0000 ± 0.0001 | 0.0052 ± 0.0016 | 0.0305 ± 0.0089 | |
|  |  | |  |  |  |  |  |  | |
| Generalized heritability^\|\|^ | 0.952 | | 0.830 | 0.841 |  | 0.906 | 0.486 | 0.602 | |

* *p* < 0.05; ** *p* <0.01; *p* < 0.00001; ns = non-significant, using a conditional Wald test.

^†^ The intercept was set to the first trial year (2003 = 0), and was averaged across maturity groups in Austria.

^‡^ For yield and protein yield: $\frac{\beta}{\mu}*100$; for protein content: based on change per year in back-transformed proportion scale.

^§^ G = genotype, L = location, Y = year, S(LY) = subtrial within location-year; M = maturity group, GLY' = combined three-way interaction + plot error.

^||^ Cullis et al. (2006): $H^{2}=1-(\frac{\bar{v}_{\Delta..}^{BLUP}}{{2\sigma}_{G}^{2}})$

*Table S2. Variance components and correlations (± SE) from the mixed models combining Austria (AT) and France (FR).*

| Component* | Protein content (logit^2^) | |  | Protein yield (t^2^ha^-2^) | |  | Grain yield (t^2^ha^-2^) | |
| --- | --- | --- | --- | --- | --- | --- | --- | --- |
|  | AT | FR |  | AT | FR |  | AT | FR |
| L | 0.0097 ± 0.0045 | 0.0057 ± 0.0014 |  | 0.0034 ± 0.0060 | 0.0538 ± 0.0150 |  | 0.0232 ± 0.0288 | 0.2718 ± 0.0748 |
| Y | 0 | 0.0009 ± 0.0006 |  | 0.0106 ± 0.0089 | 0.0032 ± 0.0038 |  | 0.0633 ± 0.0453 | 0.0256 ± 0.0220 |
| LY | 0.0155 ± 0.0022 | 0.0034 ± 0.0006 |  | 0.0937 ± 0.0141 | 0.0414 ± 0.0073 |  | 0.4135 ± 0.0632 | 0.1836 ± 0.0342 |
| S(LY) |  | 0.0000 ± 0.0001 |  |  | 0.0050 ± 0.0015 |  |  | 0.0301 ± 0.0089 |
| G | 0.0038 ± 0.0006 | 0.0048 ± 0.0009 |  | 0.0082 ± 0.0016 | 0.0022 ± 0.0011 |  | 0.0483 ± 0.0091 | 0.0343 ± 0.0139 |
| GL | 0.0000 ± 0.0000 | 0.0003 ± 0.0001 |  | 0.0010 ± 0.0003 | 0.0022 ± 0.0006 |  | 0.0062 ± 0.0018 | 0.0093 ± 0.0033 |
| GY | 0.0002 ± 0.0000 | 0.0002 ± 0.0001 |  | 0.0019 ± 0.0004 | 0.0020 ± 0.0006 |  | 0.0108 ± 0.0021 | 0.0120 ± 0.0032 |
| GLY' | 0.0015 ± 0.0001 | 0.0009 ± 0.0001 |  | 0.0132 ± 0.0005 | 0.0074 ± 0.0007 |  | 0.0692 ± 0.0028 | 0.0422 ± 0.0037 |
| MLY | 0.0015 ± 0.0003 | 0.0002 ± 0.0001 |  | 0.0086 ± 0.0018 | 0.0009 ± 0.0012 |  | 0.0452 ± 0.0095 | 0.0028 ± 0.0062 |
|  |  |  |  |  |  |  |  |  |
| $\rho_{G}$ | 0.998 ± 0.010 | |  | 0.8174 ± 0.1620 | |  | 0.7797 ± 0.1428 | |
| $\rho_{GY}$ | -0.2481 ± 0.3692 | |  | 0.6364 ± 0.3039 | |  | 0.7498 ± 0.2828 | |
| $\rho_{Y}$ | inestimable | |  | -0.6233 ± 0.6427 | |  | -0.2089 ± 0.5433 | |

*^*^* G = genotype, L = location, Y = year, GLY' = combined three-way interaction + plot error, $\rho_{.}$ = correlation.
